# Enhancing Compressive Strength of Cement by Indigenous Individual and Co-Culture *Bacillus* Bacteria

**DOI:** 10.3390/ma17204975

**Published:** 2024-10-11

**Authors:** Tiana Milović, Vesna Bulatović, Lato Pezo, Miroslav Dramićanin, Ana Tomić, Milada Pezo, Olja Šovljanski

**Affiliations:** 1 Faculty of Technical Science, University of Novi Sad, Trg Dositeja Obradovića 6, 21000 Novi Sad, Serbia; tiana.milovic@uns.ac.rs (T.M.); vesnam@uns.ac.rs (V.B.); dramicanin@uns.ac.rs (M.D.); 2 Institute of General and Physical Chemistry, University of Belgrade, Studentski Trg 12–16, 11000 Belgrade, Serbia; latopezo@yahoo.co.uk; 3 Faculty of Technology Novi Sad, University of Novi Sad, Bulevar Cara Lazara 1, 21000 Novi Sad, Serbia; 4 Department of Thermal Engineering and Energy, “Vinča” Institute of Nuclear Sciences—National Institute of the Republic of Serbia, University of Belgrade, Mike Petrovića Alasa 12–14, 11001 Belgrade, Serbia; milada@vinca.rs

**Keywords:** self-healing cement, autochthonous bacteria, compressive strength, wet–dry cycles, calcium carbonate precipitation, *Bacillus licheniformis*, *Bacillus muralis*, artificial neural networks

## Abstract

Using a Taguchi experimental design, this research focuses on utilizing indigenous bacteria from the Danube River to enhance the self-healing capabilities and structural integrity of cementitious materials. *Bacillus licheniformis* and *Bacillus muralis* were used as individual bacterium or in co-culture, with a concentration of 8 logs CFU, while the humidity variation involved testing wet and wet–dry conditions. Additionally, artificial neural network (ANN) modeling of the compressive strength of cement samples results in improvements in compressive strength, particularly under wet–dry conditions. By inducing targeted bacterial activity, the formation of calcium carbonate precipitates was initiated, which effectively sealed formed cracks, thus restoring and even enhancing the material’s strength. In addition to short-term improvements, this study also evaluates long-term improvements, with compressive strength measured over periods extending to 180 days. The results demonstrate sustained self-healing capabilities and strength improvements under varied environmental conditions, emphasizing the potential for long-term application in real-world infrastructure. This study also explores the role of environmental conditions, such as wet and wet–dry cycles, in optimizing the self-healing process, revealing that cyclic exposure conditions further improve the efficiency of strength recovery. The findings suggest that autochthonous bacterial co-cultures can be a viable solution for enhancing the durability and lifespan of concrete structures. This research provides a foundation for further exploration into bio-based self-healing mechanisms and their practical applications in the concrete industry.

## 1. Introduction

One of the remarkable innovations in concrete technology is the development of self-healing cementitious materials through the incorporation of bacterial agents. This type of bio-inspired material utilizes microbial calcite precipitation to heal cracks that develop autonomously, thereby enhancing the material’s durability and reducing maintenance costs. *Bacillus* species, especially *Bacillus pasteurii* and *Bacillus subtilis*, are commonly used because of their ability to thrive in the high pH environment of concrete and produce calcite, effectively sealing micro-cracks [1,2,3]. Indigenous bacteria, which naturally exist in specific environments, are being increasingly utilized in self-healing cement to promote calcite precipitation, a process that fills cracks and restores the material’s structural integrity [4,5]. The use of these bacteria in self-healing cementitious materials is based on microbial-induced calcium carbonate precipitation (MICP). It can be seen that different bacterial strains vary in effectiveness. For example, comparing bacterial-based types of cement to traditional mixes shows that biological components can increase strength by up to 20%, supporting sustained compressive strength over time [6]. The strength enhancement primarily occurs when calcium carbonate precipitates fill cracks and voids. For instance, MICP-enabled bacterial mortar repaired cracks 3.5 times faster than untreated samples, significantly improving strength under high loads. This self-repair is driven by bacterial activity, which facilitates the rapid formation of calcium carbonate through metabolic processes. Unlike the slower carbonation process, which occurs naturally over time, MICP accelerates crack repair by actively precipitating calcite, filling the cracks, and restoring structural integrity in a significantly shorter timeframe [7]. While bacterial inclusion can initially reduce compressive strength, it also shows long-term benefits, increasing strength by 15% after 28 days [8]. For example, among tested bacteria *P. fluorescence*, *B. pumilis*, and *B. subtilis*, the last-mentioned species provided the highest strength increase (5.92%) [9]. According to the available data, almost all research articles use only one bacterial strain, but a promising area uses the natural-coexisting phenomenon of bacterial communities. For example, in a study on geopolymer-based mortar, *Bacillus subtilis* and *Bacillus megaterium* co-cultured with polypropylene fibers significantly improved compressive strength, achieving a nearly 200% regain compared with control samples and demonstrating the potential of bacterial co-cultures for both self-healing and structural reinforcement [10]. The self-healing capacity of cementitious materials is strongly affected by environmental conditions, especially wet and wet–dry cycles. These cycles mimic the natural moisture variations concrete experiences, which can either enhance or hinder the self-healing process depending on material composition and external factors. Wet–dry cycles are crucial for activating and sustaining bacterial activity as well as the self-healing process in cementitious materials [11]. Research indicates that repeated wetting and drying improves crack healing efficiency in bio-concrete. For instance, a study using *Bacillus cibi* and lightweight aggregate (LWA) encapsulation showed that cracks up to 0.541 mm healed after 35 days of wet–dry cycling, suggesting these conditions optimize bacterial self-healing by providing moisture for microbial activity and dryness for crystallization [12].

Artificial neural networks (ANNs) are increasingly used to model and predict the behavior of self-healing cement, particularly in assessing how various factors influence compressive strength and durability. ANNs are powerful tools for optimizing self-healing concrete design by simulating the complex interactions between bacterial activity, material composition, and environmental conditions [13,14]. For example, ANNs have been employed to predict the compressive strength of bacterial concrete based on variables like bacterial concentration, curing time, and environmental factors. These models, trained on experimental data, can accurately forecast the outcomes of different self-healing cement formulations, reducing the need for extensive trials and enabling the exploration of a broader range of variables to optimize performance. ANNs have been used to predict the compressive strength of high-performance concrete (HPC) and modified concretes by training models on data from mixture components like cement, aggregates, and additives [15,16,17]. By incorporating input parameters such as bacterial type and concentration, nutrient presence, and environmental conditions, ANNs can accurately predict compressive strength, facilitating the optimization of mix designs to effectively integrate bacterial healing agents [17].

Integrating autochthonous bacterial co-cultures into cement offers a promising approach to enhancing the durability and sustainability of cement-based composites. In this study, the activation of autochthonous bacterial co-cultures over time, as well as the influence of different curing conditions on their activity for 14 days, was examined indirectly by determining the compressive strength of the paste samples at the age of 28, 60, 90, and 180 days. Additionally, the use of ANNs in this field can provide further opportunities to optimize the design and application of self-healing cement, minimizing the need for expensive experiments and improving material performance. Therefore, this study evaluates the Taguchi experimental design, kinetic modeling, and ANN modeling of the compressive strength of hardened cement paste samples using *B. licheniformis* and *B. muralis*, natural *Bacillus* species isolated from the Danube River. By accurately modeling the impact of these bacteria as individual bioagents and co-cultures, this research aims to demonstrate the potential of mathematical tools to understand bacterial contribution and predict the strength of self-healing cement. While immediate self-healing effects are often the primary focus, long-term properties are equally crucial for practical applications of self-healing cementitious materials. This study not only evaluates the initial self-healing response but also examines how these materials perform over extended periods under fluctuating environmental conditions.

## 2. Materials and Methods

### 2.1. Bacterial Strains and Co-Culturing Protocol

For this study, ureolytic bacteria from the *Bacillus* genus, *B. licheniformis* and *B. muralis*, were chosen based on their previously demonstrated high performance in MICP [4,18,19,20,21]. These bacteria were isolated from the Danube River area, additionally giving these bioagents a specific geographical origin. *B. licheniformis* and *B. muralis* strains were preserved in lyophilized form(s). The lyophilized individual cultures and co-cultures were used as an additive to the cement paste at a final concentration of 8 log CFU units per gram of cement paste. The high initial concentration of bacterial cells was chosen to ensure sufficient bacterial activity for the desired MICP effect while minimizing potential adverse effects on the material’s workability, which was reported for the bacteria used in studies by Šovljanski et al. [19,20,21].

### 2.2. Cement Sample Preparation

Cement pastes, with a water-to-cement ratio of 0.5, were prepared by (i) adding ordinary Portland cement (CEM I) to deionized water (control samples) or (ii) mixing CEM I with a lyophilized bacterial agent and then putting those dry ingredients into deionized water (bacteria samples). Deionized water was also used to avoid the influence of naturally presented microflora in tap water on the experiment. The mixing was performed in a mortar mixer set at a low speed. Subsequently, the homogenized cement paste was cast into cube molds measuring 10 mm on each side, which were then slightly vibrated for the removal of unwanted entrapped air from the pastes. The filled molds were covered with a plastic film to prevent moisture loss and left for 24 h to set. After an initial 6 h, both control and bacterial samples were further divided into two groups as follows:

(i) Intact samples without any induced defects (non-cracked samples);
(ii) Samples with an artificially created crack to simulate damage (cracked samples).

The artificial crack for cracked samples was made by a straight-edged metal blade. The blade was plunged in the middle of the upper edge to a depth of 2 mm so that the target crack size of 10 × 0.5 × 2 mm was obtained. After this initial 24 h setting period, the samples were de-molded and transferred to the containers for further curing underwater at (20 ± 1 °C) until 15 days before compressive strength testing.

### 2.3. Experimental Design

To evaluate the self-healing capabilities of the samples, an experimental setup was established using a Taguchi L4-based experimental design involving three factors (humidity variation, *B. licheniformis* concentration, and *B. muralis* concentration) and 4 runs/experiments (Table 1) and incorporating both control and bacterial-inoculated samples. For easier interpretation of the results, abbreviations were used in each experiment (C—control sample; W—wet conditions; CR—cracked sample; B_1_—*B. licheniformis*; B_2_—*B. muralis*; WD—wet–dry conditions). The testing periods lasted 28, 60, 90, and 180 days, while each sample set involved 10 repetitions (i.e., 10 of the same samples cured in the same conditions). Fifteen days before the testing period, the samples were taken out of the water, air-dried for 24 h in laboratory conditions, and then subjected to two different curing conditions to simulate environmental effects on the healing process as follows:

(i) Wet Condition: Samples were kept continuously moist by storing them in a humidity-controlled environment at 95% relative humidity; 80% of the sample height was submersed under water.
(ii) Wet–Dry Condition: Samples underwent cyclic wetting and drying. This involved 1 h of wetting followed by 11 h of drying in a controlled environment (60% humidity), repeating for the testing period.

Regardless of the humidity variations, the incubation conditions involved a temperature of 20 ± 1 °C and oxygen-depended setup, while activation of the targeted phenomenon was induced with a one-shot injection of nutrient media (100 μL) into the middle of the crack before a 14-day period at different curing conditions. The used nutrient medium consisted of urea (20 g/L), CaCl_2_ (2.12 g/L) nutrient broth (3 g/L), NiCl_2_ (20 µg/L), NH_4_Cl, (10 g/L), and NaHCO_3_ (2.15 g/L). For control samples, an injection of water was used at the same volume.

### 2.4. Compressive Strength Evaluation

**Figure 1:**
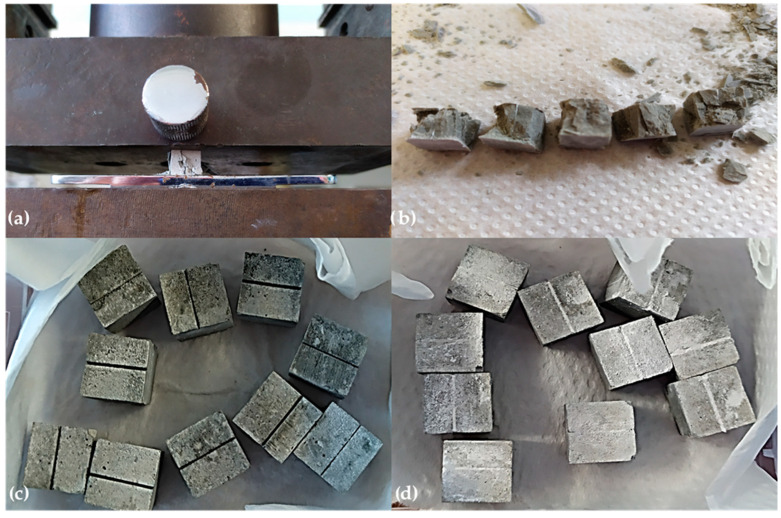
Compressive strength testing after 28 days. (**a**) Sample position on the hydraulic press. (**b**) Self-healed bacterial samples after compressive strength testing. (**c**) Cracked control samples (CCRW) prior to testing. (**d**) Self-healed bacterial samples prior to testing (B_1_B_2_CRW).

To examine the activation of bacteria over time, as well as the influence of different curing conditions on their activation and their activity over 14 days, the compressive strength of the paste samples after exposure to the self-healing process was determined. The samples were removed from their curing conditions and wiped clean of any loose material or standing water. Each sample was placed between the platens of the testing machine (Figure 1a). A Universal testing machine ZDM 5/91 (WPM, Leipzig, Germany) with a range of 25 kN was used for those purposes. The maximum load sustained by each sample before failure was recorded. The compressive strength for each sample set was determined as the average value of 10 test results. The results from the compressive strength tests were compiled and analyzed statistically to compare the performance of bacterial versus control samples under different environmental conditions. Figure 1b shows the samples after pressure setup, while Figure 1c,d present cracked control and self-healed bacterial samples prior to testing at the age of 28 days.

### 2.5. Kinetics Modeling

In this study, the kinetics of individual and co-culture *Bacillus* bacteria growth was modeled using a four-parameter sigmoidal computational model, as detailed by [4,22]. This model is well suited for biological systems, with the projected data expected to follow an S-shaped curve, as represented in Equation (1):

(1) $$y(t)=d+\frac{a-d}{1+{\left(\frac{t}{c}\right)}^{b}}$$

where *y*(*t*) represents the compressive strength (MPa) over the incubation period (hours). The regression coefficients are defined as follows: *a* is the minimum observed value (at *t* = 0), *d* is the maximum value reached (at *t* = ∞), and *c* is an inflection point (the midpoint between *a* and *d*, and *b* is Hill’s slope) indicating the steepness at the inflection point. The same model was used for non-cracked and cracked sample compressive strength modeling.

### 2.6. Artificial Neural Network Modeling

The data for ANN modeling, consisting of non-cracked and cracked samples, was divided into training (60%), cross-validation (20%), and testing (20%) sets. To enhance accuracy, min–max normalization was applied for input and output normalization. The proposed multilayer perceptron (MLP) model featured a three-layer feedforward architecture with backpropagation training [23,24]. The hidden layer contained 5 to 10 neurons, and various activation functions—tangent, sigmoidal, exponential, and identity—were tested. The Broyden–Fletcher–Goldfarb–Shanno (BFGS) algorithm was employed to develop the ANN model, iteratively adjusting weights and biases across 100,000 configurations to minimize the squared error and to bring both learning and cross-validation curves close to zero. Computations were conducted using StatSoft Statistica (version 10.0, Palo Alto, CA, USA). The model’s accuracy was assessed to predict compressive strength through several standard computational tests, including the coefficient of determination (r^2^), reduced chi-square (χ^2^), mean bias error (MBE), root mean square error (RMSE), mean percentage error (MPE), sum of squared errors (SSE), and average absolute relative deviation (AARD).

## 3. Results

The data presented in Table 2 outline the compressive strength values of both non-cracked and cracked samples over different ages, i.e., at 28, 60, 90, and 180 days. For the experimental setup, Taguchi experimental design L4 was used, and the obtained compressive strength of all samples showed a consistent increase over time across all experimental runs.

**Figure 2:**
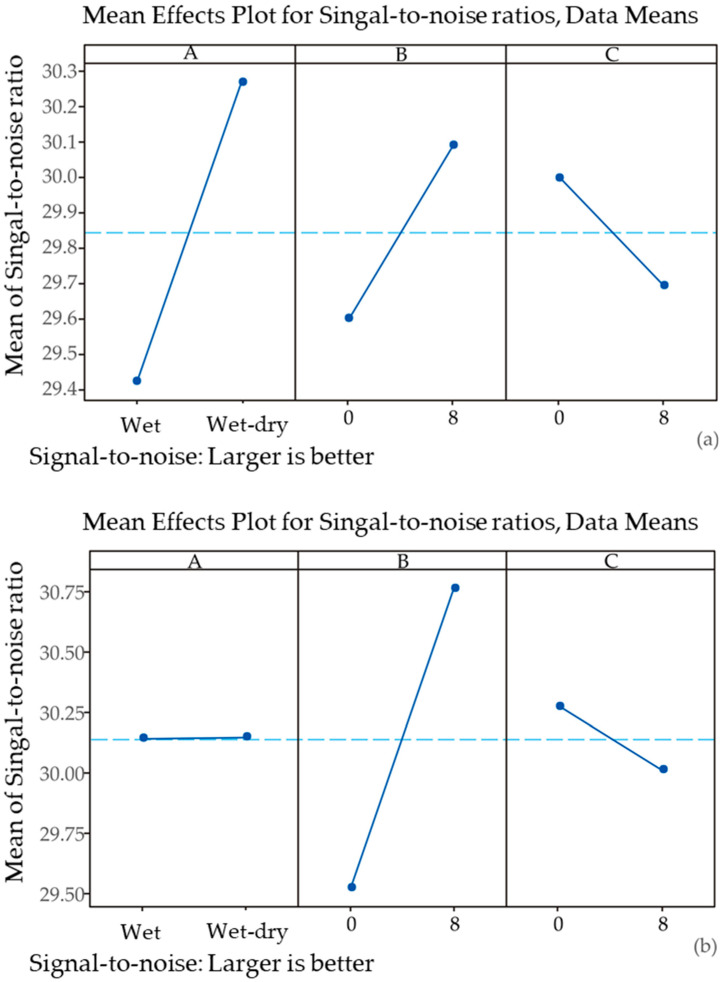
Main effects plots of the signal-to-noise (S/N) analysis of the Taguchi-obtained experimental data (A—humidity variation; B—*Bacillus licheniformis*; C—*Bacillus muralis*; for (**a**) non-cracked and (**b**) cracked samples according to the Taguchi design (see Table 1)).

The main effects plot for S/N ratios, as presented in Figure 2, provides critical insights into the influence of three factors (factors A, B, and C) on the performance of the process or system under investigation. The objective, as indicated by the plot, is to maximize the S/N ratio, with the understanding of the “*larger is better*” option.

**Figure 3:**
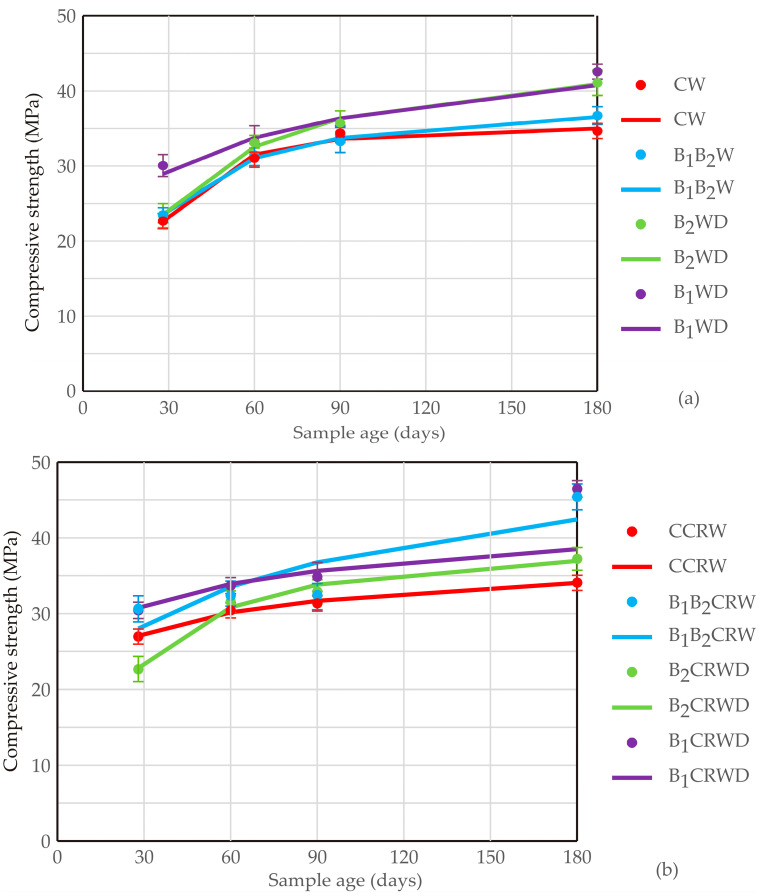
Kinetic modeling of compressive strength of (**a**) non-cracked and (**b**) cracked samples according to the Taguchi-based data (points indicate experimentally obtained results, while lines present mathematically predicted results).

The diagrams in Figure 3 illustrate the development of compressive strength (expressed in MPa) over time for various sample runs, with data points corresponding to different sample ages ranging from 28 to 180 days. The results reveal a clear trend across all samples, where compressive strength generally increases as the material ages. This behavior is consistent with typical materials such as concrete, which gain strength over time because of the hydration process. Except for short-term healing, the compressive strength results demonstrate significant long-term improvements. Over 180 days, samples subjected to wet and wet–dry cycles showed consistent gains in compressive strength, highlighting the durability of the self-healing mechanism. Based on these results, it can be suggested that the autochthonous bacterial co-cultures contribute to immediate crack healing and continue to enhance material integrity well into the long term, making them viable for real-world applications where durability is a priority.

The compressive strength results reveal that both non-cracked and cracked samples benefited from the addition of bacterial co-cultures, with distinct differences between the two. Non-cracked samples demonstrated a continuous and steady increase in compressive strength over time, with bacterial co-cultures further enhancing the strength. The self-healing process in cracked samples led to a strength regain compared with control cracked samples, highlighting the effectiveness of bacterial-induced calcium carbonate precipitation in restoring structural integrity.

The data in Table 3 present the verification results of the kinetic models based on several quality parameters across different runs. These parameters include statistical measures such as the chi-square statistic (*χ*^2^), root mean square error (RMSE), mean bias error (MBE), mean percentage error (MPE), sum of squared errors (SSE), average absolute relative deviation (AARD), along with additional statistical characteristics such as the coefficient of determination (*r*^2^), skewness (Skew), kurtosis (Kurt), mean, standard deviation (StDev), and variance (Var). Furthermore, verification refers to comparing the model-predicted values with the actual experimental data to assess the accuracy and reliability of the kinetic models. The table includes important statistical parameters such as RMSE and *r*^2^ values, which are used to evaluate the goodness of fit between the predicted and observed data. *r*^2^ values close to 1 indicate a strong correlation between the model predictions and the experimental results, while lower RMSE values reflect minimal deviations between the two, suggesting that the kinetic models accurately capture the behavior of the self-healing process.

The data in Table 4 present the performance metrics of the ANN models applied to both non-cracked and cracked samples. The table includes information on the network architecture, training, testing, and validation performance, as well as error metrics and additional statistical characteristics that evaluate the model’s accuracy and reliability. The network model utilized for non-cracked samples was an MLP 5-3-1, consisting of five input nodes, three hidden nodes, and one output node. The training performance of this model is reported as 0.878, while both the test and validation performances are perfect, recorded at 1.000. Despite this high performance, the training error is 1.589, and the test and validation errors are higher, at 1.706 and 4.477, respectively. The network training was conducted using the BFGS 24 algorithm, with a sum of squares (SOS) error function. The hidden layer activation function was exponential, while the output activation function was logistic. Regarding verification, the ANN model for non-cracked samples displays a chi-square (χ^2^) value of 4.191 and an RMSE of 1.982, indicating moderate model accuracy. The MBE and MPE are 0.429 and 3.892, respectively, which suggests some bias and percentage error in the model’s predictions. Additionally, the SSE is 62.872, and the AARD is 3.892, further reflecting the moderate error level. The *r*^2^ value for this model is 0.877, which indicates a strong correlation between the observed and predicted values, though not perfect. The skewness and kurtosis are 0.964 and 0.987, respectively, suggesting a slight positive skew and near-normal distribution of errors. The mean error is 0.429, with a standard deviation of 1.999 and a variance of 3.995, indicating some variability in the error distribution. For cracked samples, the network model was an MLP 5-5-1, comprising five input nodes, five hidden nodes, and one output node. The training performance is 0.837, while the test and validation performances are reported as perfect at 1.000. However, the training error is higher compared with non-cracked samples, at 2.440, with test and validation errors of 1.108 and 12.153, respectively. This network was trained using the BFGS 19 algorithm, again with an SOS error function. Both the hidden and output activation functions were tangent hyperbolic. The verification metrics for the ANN model applied to cracked samples show a higher chi-square (χ^2^) value of 7.440 and a higher RMSE of 2.641, indicating less accuracy compared with the non-cracked model. The MBE is 0.558 and the MPE is 5.976, which suggests a higher bias and percentage error in the predictions. The SSE is also notably higher at 111.606, and the AARD is 5.976, reflecting greater errors and inconsistencies in the model predictions. The *r*^2^ value for cracked samples is 0.810, indicating a weaker correlation between observed and predicted values than that of non-cracked samples. The skewness is 0.946, and the kurtosis is 0.716, indicating a moderate positive skew and slightly leptokurtic distribution, with the mean error at 0.558. The standard deviation is 2.666, and the variance is 7.108, pointing to greater variability in the error distribution compared with non-cracked samples.

In summary, Table 4 presents the results of the ANN model, which predicts the compressive strength of self-healing cement paste samples. The table includes data about the ANN models and ANN verification parameters. These metrics help assess the accuracy and reliability of the ANN model in capturing the effects of bacterial activity and environmental conditions on compressive strength. The high *r*^2^ values indicate a strong correlation between the predicted and actual values, confirming that the ANN model provides accurate predictions for the self-healing performance of cement-based materials.

**Figure 4:**
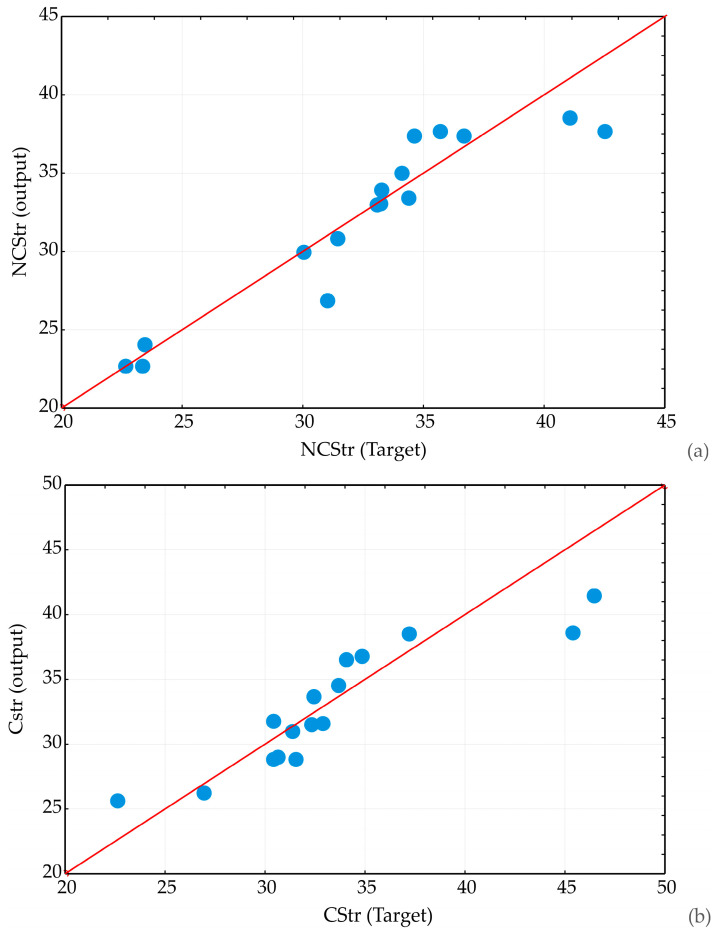
Scatter plots of correlations between target compressive strength and predicted compressive strength for (**a**) non-cracked (NCS) samples and (**b**) cracked (CS) samples.

The provided scatter plots (Figure 4) illustrate the relationship between the target compressive strength and the predicted compressive strength across various samples, which include training, testing, and validation datasets. The plot includes a red line representing the ideal scenario where the predicted values perfectly match the target values (i.e., the line of equality where NCStr Target equals NCStr Output or CStr Target equals CStr Output).

## 4. Discussion

The use of the Taguchi experimental design, complemented by signal-to-noise S/N analysis, is crucial for systematically optimizing the factors influencing the compressive strength of self-healing cement pastes. A similar approach was used in the study by Abdurrahman and Putra [25], where Taguchi methods were utilized to optimize parameters for self-healing concrete, particularly focusing on *Bacillus subtilis.* This approach can ensure efficient experimentation and lead to more robust, reliable, and high-quality outcomes [26], which are essential for the successful application of self-healing cement in the concrete industry. According to the data obtained after the evaluation of the Taguchi experimental design (Table 2), control non-cracked samples started with a compressive strength of 22.65 MPa at 28 days and gradually increased to 34.64 MPa by 180 days. On the other hand, non-cracked samples with individual bacterium had the highest initial strength of 30.05 MPa at 28 days and achieved the highest compressive strength of 42.54 MPa at the end of the testing period. This trend indicates a progressive enhancement in the material’s strength as it ages. The standard deviation across these runs was relatively small, especially at later stages, suggesting consistent performance in non-cracked samples over time. In contrast, cracked samples exhibited a different pattern. These samples generally began with higher initial compressive strengths at 28 days compared with non-cracked samples. For example, control cracked samples started with a compressive strength of 26.97 MPa, while cracked samples with *B.licheniformis*–*B.muralis* co-culture started at 30.63 MPa. By 180 days, the compressive strength of cracked samples also showed significant increases, where samples with *B. licheniformis* reached the highest value of 46.45 MPa. Comparing the two sample types, it is evident that cracked samples tend to start with equal or higher compressive strengths at earlier ages but demonstrate more variability in performance. On the other hand, non-cracked samples, despite starting with lower compressive strengths, show a more consistent and steady increase in strength over time, especially at later stages. Furthermore, cracking might lead to higher early-age strength, but non-cracked samples provide more reliable and consistent long-term strength development. One more observation can be directed to CW and CCRW samples at 28 days (Table 2). The observed difference in compressive strength between CW and CCRW at 28 days can be explained by the autogenous self-healing mechanism in cracked samples. While the CW mixture did not benefit from that mechanism, the CCRW mixture likely experienced enhanced hydration and micro-crack (that were obtained during the formation of the artificial crack 6 h from preparation and molding of the cement paste mixture) closure due to water penetration into the cracked regions. This led to the reformation of hydration products, which filled the micro-cracks and improved the overall structural integrity of the CCRW samples. As a result, cracked samples displayed higher compressive strength compared with non-cracked CW samples at the age of 28 days.

For a deeper analysis of Taguchi-based data, S/N analysis was conducted. Based on Figure 2a, the analysis of humidity variation for non-cracked samples revealed a significant positive effect on the S/N ratio as this factor changed from wet to wet–dry conditions. The marked increase in the S/N ratio suggests that wet–dry conditions are strongly associated with improved performance, making it a key factor in optimizing the process. Similarly, *B. licheniformis* presence also showed a positive influence on the S/N ratio, though the effect was less pronounced compared with humidity conditions. The increase in the S/N ratio from 0 to 8 log CFU for *B. licheniformis* indicates that higher levels of this factor contribute to better performance, albeit to a lesser extent than wet–dry cycles. In contrast, *B. muralis* exhibited a different behavior. The S/N ratio decreased as the level of *B. muralis* increased from 0 to 8 log CFU. This inverse relationship suggests that higher levels of *B. muralis* negatively impact the performance, making it less desirable to increase this factor. It can be concluded that the main effects plot for non-cracked samples (Figure 2a) demonstrates that wet–dry conditions and the presence of *B. licheniformis* positively influence the compressive strength of non-cracked cement paste samples, with the most substantial impact of hydration variations in the system. For optimal performance based on S/N ratios, the results suggest that it is advantageous to involve wet–dry cycles as well as *B. licheniformis* concentration while either decreasing or maintaining *B. muralis* at a lower level. The same analysis of the S/N ratio was performed for cracked samples (Figure 2b). The main effects plot for S/N ratios in the provided figure highlights the effects of targeted factors on the performance metric. For humidity variation, the plot shows a minimal effect on the S/N ratio, with the mean of S/N ratios remaining nearly constant between wet and wet–dry conditions. This indicates that changes in humidity do not significantly impact the compressive strength of cracked samples, suggesting that this factor may have a negligible influence in the context of the process or system being studied. In contrast, *B. licheniformis* displays a substantial positive impact on the S/N ratio. The S/N ratio increases significantly when the bacterium is present in the samples, indicating that higher concentrations of *B. licheniformis* are associated with an improved performance of cement paste samples. Furthermore, the explained influence can involve the hypothesis that the presence of *B. licheniformis* in cement can be a critical factor in inducing the self-healing phenomenon, and increasing its level could lead to better outcomes.

Based on the experimentally obtained results and the mathematical analysis, the presence of *B. licheniformis-B. muralis* co-culture leads to enhanced compressive strength in self-healing cement samples. This improvement can be primarily related to MICP, where bacteria facilitate the formation of calcite that fills micro-cracks and strengthens the material. Bacterial samples demonstrate a significant self-healing ability due to the active role of bacteria in precipitating calcium carbonate, which repairs cracks and maintains the structural integrity of the cement paste. This healing capability is especially noticeable under environmental conditions that favor bacterial activity, such as wet–dry cycles. The presence of the used bacteria in the cement matrix enhances the material’s durability, particularly under harsh environmental conditions such as fluctuating moisture levels. Bacterial activity in self-healing cement paste is influenced by a variety of environmental factors, including temperature, humidity, and pH levels, all of which can impact both short-term and long-term performance [4]. While this study focuses on the effects of humidity through wet and wet–dry cycles, it is essential to explore the influence of temperature and pH in future research to understand all the environmental parameters that affect bacterial activity and cement paste performance.

The observed performance of non-cracked and cracked samples demonstrates how bacterial co-cultures influence both the continuous improvement in intact materials and the recovery of damaged structures. Non-cracked samples showed a steady increase in compressive strength over time, largely due to the sustained bacterial activity that contributes to the overall densification of the cement matrix through calcium carbonate precipitation. On the other hand, B_2_CRWD cracked samples experienced a significant initial strength reduction at the age of 28 days. However, the bacterial co-cultures facilitated an efficient self-healing process, particularly under wet–dry cycles, where calcium carbonate precipitates filled the cracks. Biological processes help maintain the material’s integrity, leading to a longer lifespan. The application of bacterial co-cultures, where different strains of bacteria are combined, has been shown to significantly improve the efficiency of calcite precipitation, which is critical for the self-healing process. Only a few recent studies that deal with this topic highlight the effectiveness of bacterial co-cultures in MICP for self-healing materials [27]. The synergistic potential of co-culture of bacterial strains can lead to strength and durability in cementitious materials, making them more resilient to environmental stresses and mechanical damage. The integration of ureolytic and non-ureolytic bacteria, as discussed by Son et al. [28], further improves the rate and extent of calcium carbonate precipitation. Additionally, Noshi and Schubert [29] provided evidence that bacterial co-cultures in MICP can significantly enhance the mechanical properties of cement-based materials, particularly in challenging conditions. Regarding similar studies, only a recent study conducted by Bark et al. [30] involved two *Bacillus* species for improving the mechanical properties of cement-based materials. They found that the use of a co-culture of *B. megaterium* MTCC 1684 and *B. subtilis* NCIM 2193 positively contributed to enhanced compressive strength and crack closure rates. On the other hand, their study involved reference strains (obtained from commercial culture collections), while this study elaborated using specific, autochthonic strains isolated from a specific geographic area, i.e., the Danube River. Based on the obtained results, the co-culture approach can accelerate the self-healing process and ensure more comprehensive crack repair in concrete structures.

In the next step, kinetic modeling was performed to compare the experimentally obtained and mathematical predicted results. Among all kinetics models for non-cracked samples (Figure 3a), the experiment that involved bacteria exhibited the most significant increase in compressive strength, starting between 23 and 30 MPa at 28 days and reaching above 35 MPa at 180 days. This suggests that the conditions and material composition used in this experiment are particularly effective in enhancing strength over time, thus predicting that they are the most promising for applications requiring long-term durability and strength. Applying only one bacterial strain also shows strong performance, with compressive strength steadily increasing to about 40 MPa by 180 days. Although slightly lower than the synergistic effect of *Bacillus* species, the steady growth indicates that using individual bioagents also benefits from favorable conditions, contributing to its robust strength development. In contrast to the S/N ratio analysis, the kinetics models reveal that the presence of *B. licheniformis* exhibits a more moderate increase in compressive strength, leveling off at approximately 35 MPa after 90 days. This plateau suggests that the material reached its maximum potential strength earlier than other bacteria-involved samples, indicating a potential limitation in the factors influencing this sample’s performance. However, control samples (no influence of bacteria) demonstrated the lowest compressive strength throughout the aging process. After an initial rise to about 30 MPa at 60 days, the strength plateaus, showing minimal further improvement.

The kinetic models in Figure 3b present the compressive strength of cracked samples over time plotted against the sample age. The data indicate that all samples exhibit an increase in compressive strength as they age, which is consistent with the expected behavior of materials like concrete that continue to gain strength over time. However, there are notable differences in the rate and extent of strength development among the different runs. Using bacteria to induce the self-healing performance of cracked samples demonstrates the highest overall performance, which correlated with the S/N analysis of the Taguchi data (Figure 2a). Starting at a moderate strength of around 30 MPa at 28 days, this sample shows a steady and continuous increase in compressive strength, reaching approximately 45 MPa by 180 days. This consistent upward trend suggests that the presence of co-culture is highly conducive to long-term strength development. Cracked samples that involved individual species follow similar patterns, although their rate of strength increase is somewhat less pronounced. Interestingly, the presence of *B. muralis* leads to a significant increase in strength by 60 days, after which the strength gain slows down considerably, plateauing at around 35 MPa. This suggests that cracked samples initially benefit from *B. muralis* activity, while the bacterial potential for further strength development may be limited. This can be an additional explanation for the obtained S/N ratios for this factor (Figure 2b). The evaluation of compressive strength over 180 days emphasizes the importance of long-term performance in self-healing materials. The consistent increase in strength, particularly in samples subjected to wet–dry cycles, indicates that the bacterial activity remains effective well beyond the initial stages of healing. This aligns with the behavior of concrete in real-world settings, where materials are often exposed to fluctuating environmental conditions. By demonstrating that self-healing processes continue to function under these conditions over extended periods, this study confirms the suitability of bio-based self-healing cement for use in infrastructure projects where long-term performance is paramount.

The analysis in Table 3 highlights significant differences in the quality of kinetic model predictions across the runs. Most experiments demonstrate superior model performance, as evidenced by their low error metrics (χ^2^, RMSE, SSE, and AARD), high *r*^2^ values, and more normal distribution of residuals (as suggested by skewness and kurtosis). Interestingly, experiments with incorporated *Bacillus* bacterium have high r^2^ values, indicating strong correlations between the observed and predicted values and excellent model fit. Based on the verification of the kinetic models (Table 3), experiments with *B. licheniformis* show the lowest χ^2^ values (0.060 and 0.143, respectively), indicating a good fit of the kinetic model with minimal deviation between the observed and predicted values. These runs also exhibit the lowest RMSE values (0.212 and 0.327), further supporting the accuracy of the model in these cases. The mentioned kinetic models exhibit the lowest SSE values (0.180 and 0.429) and low AARD values (0.582 and 0.869), indicating that the residuals (errors) are minimal and the model predictions are close to the observed values. Additionally, most runs exhibit low MBE and MPE values close to zero, indicating minimal bias in the model’s predictions. In contrast, a slightly lower fitting of kinetic models is recognized in experiments with *B. licheniformis*-*B. muralis* co-culture since slightly higher error metrics and lower *r*^2^ values are observed. In summary, the results presented in Table 3 demonstrate the accuracy of the kinetic models in predicting the compressive strength and self-healing efficiency of bacterial co-cultures in cement-based materials. The verification process, as shown in Table 3, involves comparing the predicted values from the models with the actual experimental data. The high *r*^2^ values indicate a strong correlation between the model outputs and the observed results, confirming the reliability of the models in predicting long-term performance. Additionally, the low RMSE values indicate minimal prediction errors, further validating the models’ suitability for capturing the self-healing process over time. This verification step is critical to ensure that the models can be used confidently in future studies or practical applications where accurate predictions of material performance are essential.

The analysis in Table 4 indicates that the ANN models for non-cracked samples outperform those for cracked samples in terms of both predictive accuracy and error metrics. The non-cracked sample model (MLP 5-3-1) demonstrates lower training and testing errors, stronger correlation (higher *r*^2^) and less variability in the error distribution. In contrast, the cracked sample model (MLP 5-5-1) shows higher errors, lower *r*^2^, and greater variability, indicating that the model struggles more with predicting outcomes accurately for cracked samples. These results suggest that the ANN model training process may require improvement, particularly for cracked samples, to achieve better predictive performance and reliability.

For non-cracked samples, the ANN model demonstrated excellent predictive accuracy, with high *r*^2^ values and low root mean square error (RMSE) values, indicating minimal deviations between predicted and actual compressive strength values. Non-cracked samples exhibited consistent strength gains over time due to the ongoing activity of the bacterial co-cultures, which led to continuous calcium carbonate precipitation and matrix densification. The ANN model captured these trends effectively, as the bacterial activity had a steady and positive impact on the compressive strength of non-cracked cement paste samples. In comparison, the ANN model for cracked cement samples also performed well, but with slightly lower *r*^2^ values than for non-cracked samples. This difference is likely due to the more complex nature of the healing process in cracked samples, where bacterial activity is primarily focused on healing the induced damage through calcium carbonate precipitation within the cracks. The ANN model was still able to predict the recovery of compressive strength in cracked samples, but with more variability, as the healing efficiency was influenced by factors such as crack size, environmental conditions (e.g., wet and wet–dry cycles), and the rate of bacterial activity. Despite these challenges, the ANN model accurately reflected the healing trends, demonstrating that bacterial co-cultures can effectively restore strength to cracked cement paste samples over time. The comparison between non-cracked and cracked sample predictions highlights the robustness of the ANN models in capturing the effects of bacterial self-healing in different conditions. For non-cracked samples, where the bacterial activity contributes to overall matrix strengthening, the model’s high predictive accuracy indicates a strong correlation between experimental and predicted results. In cracked samples, the ANN model still performed well, but the inherent variability in the healing process led to slightly lower accuracy. Nevertheless, the overall low RMSE values for both non-cracked and cracked samples demonstrate that the models provide reliable predictions across different conditions. The ANN model’s ability to predict the recovery of compressive strength in both non-cracked and cracked samples underscores its value as a tool for forecasting the long-term performance of bacterial self-healing cement. By integrating a broader range of experimental data, including different crack sizes and environmental conditions, future iterations of the model could further improve accuracy and applicability in real-world scenarios. Since bacterial behaviors, both the proliferation process and metabolic activities can be subjected to strong changes in cement matrix environments, the obtained predicted capacity of ANN models is expected. ANNs are highly effective in modeling the complex interactions between the microbiological aspects of self-healing cement and its resultant mechanical properties. By integrating various input parameters such as bacterial concentration and environmental conditions, ANNs can accurately predict the compressive strength and self-healing efficiency of bacterial-containing cement. The obtained ANNs can model how different bacterial strains and their metabolic activities contribute to the overall compressive strength of the cement paste. Practically, this approach allows further optimization of the bacterial activity in the mix design to maximize the material’s strength. The main differences between samples containing bacteria and controls lie in the enhanced compressive strength and superior self-healing capability.

ANNs play a critical role in modeling these differences by predicting the impact of microbial activity on the properties of cement-based materials, optimizing the mix design, and understanding the underlying mechanisms that drive the self-healing process. Recent studies have demonstrated the effectiveness of ANNs in predicting the mechanical properties of bacterial self-healing concrete. For instance, Fahimizadeh et al. [31] utilized non-ureolytic bacteria encapsulated in alginate hydrogel capsules to promote self-healing in cement paste and mortar. Their study showed significant flexural strength regain and full crack closure under wet–dry cycles, and ANN modeling was used to predict these properties with high accuracy. Their work highlights the potential of ANNs in accurately predicting the compressive strength and healing efficiency of bacterial self-healing cement under various environmental conditions. Furthermore, Hussein et al. [32] explored the use of *Bacillus subtilis* in self-healing concrete, with ANNs employed to model the compressive strength over time. The ANN model provided accurate predictions, demonstrating that this approach can be effectively used to optimize the dosage of bacterial agents and curing conditions to achieve maximum strength recovery. These examples demonstrate the capability of ANNs in enhancing the design and application of bacterial self-healing cement-based materials, providing a valuable tool for predicting bacterial role in material structures.

The results from the ANN model indicate that it is highly accurate within the scope of the experimental data, which focused on humidity variations and bacterial concentrations. The model showed strong predictive capability, as demonstrated by high *r*^2^ values and low RMSE and MBE. It can be concluded that the ANN approach is a powerful tool for predicting compressive strength in self-healing cement paste under controlled conditions. However, the generalization of the model is currently limited by the range of conditions under which it was trained and tested. The current dataset does not account for variations in other environmental factors, such as temperature and pH, which are known to influence bacterial activity and, consequently, the self-healing process. As a result, the model’s performance under more diverse environmental conditions has not yet been fully validated. Future work will focus on collecting additional data across a wider range of temperatures and pH levels to expand the training dataset and improve the model’s robustness and generalization capability. The inclusion of these broader conditions would allow for the development of a more versatile predictive tool capable of optimizing self-healing cement paste formulations for various climates and environments.

Additionally, the majority of the data points cluster around the red line shown in Figure 4a, indicating a generally strong correlation between the predicted compressive strengths and the actual target values. This suggests that the model is reasonably successful in capturing the underlying patterns in the data for non-cracked samples, leading to accurate predictions. A few data points deviate significantly from the red line, particularly at lower and higher ranges of compressive strength. These deviations indicate instances where the model’s predictions are less accurate, either underestimating or overestimating the true compressive strength. The overall spread of data points suggests that the model performs well for moderate values of compressive strength, where most predictions closely align with the target values. However, the larger deviations at the extremes (lower and higher values) suggest that the model might need further refinement to improve its accuracy across the full range of compressive strengths. The proximity of many points to the line of equality reflects good model performance in the middle range of compressive strengths. However, the observed outliers indicate potential areas where the model’s predictive power could be enhanced, particularly for cases where the compressive strength is either very low or very high. The scatter plot indicates that the model used to predict compressive strength performs adequately, especially for mid-range values (around 30–35 MPa), as evidenced by the concentration of data points near the line of equality. The following scatter plot in Figure 4b shows the relationship between the target compressive strength and the predicted compressive strength for cracked samples, including the training, testing, and validation datasets. The scatter plot shows a strong correlation between the predicted compressive strengths and the actual target values for most data points, as many points are closely aligned with the red line. This suggests that the model is generally accurate in predicting compressive strength. Similar to the previous plot, some data points deviate from the red line, indicating discrepancies between the predicted and actual values. These deviations are more noticeable at the higher target compressive strength range (above 40 MPa). At mid-range values (around 30 to 35 MPa), the predictions are more accurate, with most points clustering near the red line. However, at lower target values (around 25 MPa), there is still some deviation. The concentration of points near the red line for mid-range target values indicates that the model performs well in this range, with relatively accurate predictions. However, the larger discrepancies at both the lower and upper ends of the compressive strength range suggest that the model’s accuracy diminishes at these extremes. The scatter plot demonstrates that the model used for predicting compressive strength is reasonably accurate, particularly for mid-range target values. The observed discrepancies at higher values (above 45 MPa) suggest that the model could benefit from further calibration or enhancement to improve its predictive accuracy across the full range of compressive strengths, particularly at the extremes.

As summarized by the obtained results in this study, the alternation between wet and dry conditions also promotes better cement paste sample integrity. During the wet phase, water facilitates the transport of bacterial nutrients and the precipitates formed by bacterial activity, while the dry phase allows for the crystallization and hardening of these precipitates, effectively sealing cracks. The cycle between hydration and dehydration leads to more effective crack closure than continuous wet conditions alone. The effectiveness of wet–dry cycles in enhancing the self-healing capabilities of cement paste samples with *Bacillus* species can be closely linked to the spore formation and activation processes of these bacteria. *Bacillus licheniformis* and *Bacillus muralis* are well-known for their ability to form endospores, which are highly resistant to environmental stresses [20,21]. These spores can remain dormant under unfavorable conditions and reactivate when the environment becomes conducive to growth and metabolic activity. *Bacillus* species such as *B. licheniformis* and *B. muralis* undergo sporulation in response to nutrient reduction, dehydration, or other stressors. During this process, the bacterial cell forms a highly resistant spore that can survive extreme conditions, including the desiccation associated with the dry phase of wet–dry cycles. When the environmental conditions improve, such as during the wet phase of wet–dry cycles, these spores can germinate and become metabolically active again. This reactivation allows the bacteria to resume their metabolic functions, including the production of calcium carbonate through the MICP process, which is essential for the self-healing process in cement-based materials. Wet–dry cycles mimic the natural environmental conditions that *Bacillus* spores encounter in their natural habitats. The dry phase induces sporulation, ensuring that the bacterial cells can survive in a dormant state during periods of desiccation. When the environment shifts back to wet conditions, these spores germinate, and the bacteria become active, initiating the self-healing process. The cyclical nature of wet–dry conditions thus keeps the bacteria in an active cycle of dormancy and reactivation, optimizing the periods when calcium carbonate production is most needed for healing. In continuous wet conditions, while bacteria may remain active, the lack of desiccation stress means that sporulation is less likely to occur. This could lead to a decline in the bacterial population over time as cells may become metabolically exhausted without the benefit of the recovery period provided by dry phases. Wet–dry cycles, by contrast, allow the bacterial population to periodically “rest” and then reactivate, maintaining a more consistent and robust level of bacterial activity over time, which is beneficial for long-term self-healing. The superior performance of self-healing cement paste under wet–dry cycles can indeed be correlated with the sporulation and activation processes of *Bacillus* species. Wet–dry cycles create an optimal environment that triggers the formation of spores during dry phases and their subsequent activation during wet phases. This cyclical process ensures that the bacterial population remains viable and active over extended periods, allowing for continuous production of calcium carbonate, which is crucial for the self-healing of cracks in the cement matrix. Correlating the humidity variation in the samples, wet–dry cycles provide the necessary environmental prompts for *Bacillus* spores to undergo a cycle of dormancy and reactivation, thereby enhancing their ability to contribute to the self-healing process. This cyclical activation likely explains the observed improvements in self-healing efficiency and compressive strength in cement paste samples compared with those subjected to constant wet conditions.

This finding is consistent with the work by De Muynck et al. [33], which demonstrated that repeated wetting and drying not only accelerated the microbial-induced calcite precipitation (MICP) process but also resulted in more effective crack closure compared with samples cured under continuous wet conditions. Wet–dry cycles mimic the natural environmental fluctuations that cement-based structures often experience, particularly in outdoor settings. These cycles promote the reactivation of bacterial spores during wet phases, leading to the resumption of metabolic activity that is critical for the production of calcium carbonate, which fills and seals cracks. During the dry phases, the calcium carbonate precipitates harden, effectively strengthening the material and preventing further crack propagation. Moreover, Sumathi et al. [34] demonstrated that the alternation between wet and dry conditions may enhance the penetration of nutrients and oxygen into the cement matrix, further supporting bacterial growth and activity. This cyclical environmental stress appears to optimize the conditions for MICP, making it a vital factor in the performance of self-healing cement. As observed in this study, samples subjected to wet–dry cycles showed a more substantial increase in compressive strength over time compared with those maintained under constant wet conditions, with the co-culture of *Bacillus licheniformis* and *Bacillus muralis* demonstrating particularly notable results.

## 5. Conclusions

This research investigated the integration of autochthonous bacterial individual cultures and co-cultures, specifically *Bacillus licheniformis* and *Bacillus muralis*, into self-healing cement paste, assessing their impact under various environmental conditions, particularly wet–dry cycles. The co-culture approach proves more effective than individual bacterial cultures because of the synergistic interaction between the bacterial strains. However, while the co-culture method offers notable advantages, such as enhanced crack sealing and improved strength, it also introduces complexity in managing the interaction between different bacterial species, which may require more precise control over environmental conditions to ensure optimal performance. This study not only highlights the immediate self-healing properties of autochthonous bacterial co-cultures but also confirms their long-term performance. The results, obtained over 180 days under varying environmental conditions, demonstrate sustained improvements in compressive strength. Wet–dry cycles are more effective than continuous wet cycles in promoting the self-healing capacity of *Bacillus licheniformis-Bacillus muralis* co-culture-based cement. Wet–dry cycles mimic natural environmental conditions that optimize bacterial sporulation and subsequent reactivation, ensuring that bacteria remain viable and active, maintaining their ability to heal cracks over extended periods. The application of ANNs in this study proved to be a valuable tool for modeling and optimizing self-healing cement formulations. By accurately predicting the compressive strength and healing efficiency based on various input parameters, including bacterial concentration, curing conditions, and environmental factors, ANNs facilitated the optimization of the experimental design. This approach reduces the need for extensive experimental trials, saving time and resources while improving the overall performance of the self-healing cement. This study opens several paths for future research, including the exploration of other indigenous bacterial strains that may offer even greater self-healing capabilities. Additionally, further improvement in the ANN models could provide more precise predictions and optimizations, potentially leading to the development of new formulations and applications for self-healing cement. The integration of indigenous *Bacillus* co-cultures into self-healing cement represents a promising innovation in materials science, offering enhanced compressive strength, potential performance improvements, and a more sustainable approach.

## Figures and Tables

**Table 1:** The Taguchi design for self-healing evaluation.

**Independent Variable**		**Coded** **Symbol**	**Factor Variations**	
			**Lower Level**	**Higher Level**
Factor A: Humidity variation		*X*_1_	wet	wet–dry
Factor B: *B. licheniformis* (log CFU)		*X*_2_	0	8
Factor C: *B. muralis* (log CFU)		*X*_3_	0	8
**Dependent variables**		**Coded** **Symbol**	**Constraints**	
Compressive strength (MPa)		*Y*_1_	“*Larger is better*” (Maximize value)	
**Experimental design using the coded form**				
**Run**	***X*_1_**	***X*_2_**	***X*_3_**	
CW/CCRW	wet	0	0	
B_1_B_2_W/B_1_B_2_CRW	wet	8	8	
B_2_WD/B_2_CRWD	wet–dry	0	8	
B_1_WD/B_1_CRWD	wet–dry	8	0	

Legend: C—control; W—wet conditions; CR—cracked; B_1_—*B. licheniformis*; B_2_—*B. muralis*; WD—wet–dry conditions.

**Table 2:** Taguchi experimental design-related values for compressive strength (results are presented as mean and standard deviation; repetitions of each sample type were 10).

Compressive Strength (MPa)					
Non-cracked samples					
Age (days)		28	60	90	180
Run	CW	22.65 ± 3.25 ^aA^	31.02 ± 2.47 ^aB^	34.37 ± 2.78 ^aBC^	34.64 ± 0.58 ^aC^
	B_1_B_2_W	23.46 ± 3.47 ^aA^	31.44 ± 3.91 ^aB^	33.25 ± 3.14 ^aB^	36.70 ± 3.13 ^bB^
	B_2_WD	23.37 ± 2.23 ^aA^	33.10 ± 2.76 ^aB^	35.72 ± 3.75 ^aB^	41.07 ± 1.51 ^cC^
	B_1_WD	30.05 ± 1.60 ^bA^	33.21 ± 2.85 ^aA^	34.11 ± 3.94 ^aA^	42.54 ± 1.00 ^cB^
Cracked samples					
Age (days)		28	60	90	180
Run	CCRW	26.97 ± 2.17 ^bA^	30.41 ± 1.96 ^aB^	31.35 ± 1.53 ^aB^	34.10 ± 2.84 ^aB^
	B_1_B_2_CRW	30.63 ± 3.08 ^cA^	32.36 ± 3.26 ^aA^	32.44 ± 1.14 ^aA^	45.39 ± 3.63 ^bB^
	B_2_CRWD	22.65 ± 1.71 ^aA^	31.57 ± 2.07 ^aB^	32.89 ± 2.65 ^aB^	37.23 ± 1.29 ^aC^
	B_1_CRWD	30.44 ± 3.08 ^cA^	33.70 ± 2.41 ^aA^	34.85 ± 2.99 ^aA^	46.45 ± 2.87 ^bB^

Values within a column marked by the same lowercase letter indicate that the means of different sample runs were not significantly different at the *p* < 0.05 level, according to Tukey’s HSD test. Values within a row marked by the same uppercase letter indicate that the means of different sample ages were not significantly different at the *p* < 0.05 level, also based on Tukey’s HSD test.

**Table 3:** Verification of kinetics models (quality parameters).

Run	*χ* ^2^	RMSE	MBE	MPE	SSE	AARD
Non-cracked samples						
CW	0.324	0.493	0.001	1.279	0.972	1.279
B_1_B_2_W	0.143	0.327	−0.001	0.869	0.429	0.869
B_2_WD	0.257	0.439	−0.001	1.111	0.770	1.111
B_1_WD	3.300	1.573	0.029	4.068	9.900	4.068
Cracked samples						
CCRW	0.060	0.212	0.000	0.582	0.180	0.582
B_1_B_2_CRW	11.862	2.983	0.034	8.016	35.586	8.016
B_2_CRWD	0.500	0.612	−0.002	1.638	1.500	1.638
B_1_CRWD	21.244	3.992	1.666	5.223	63.733	5.223
Run	*r* ^2^	Skew	Kurt	Mean	StDev	Var
Non-cracked samples						
CW	0.990	1.130	0.493	0.001	0.569	0.324
B_1_B_2_W	0.995	−0.620	0.073	−0.001	0.378	0.143
B_2_WD	0.995	−0.346	−0.089	−0.001	0.507	0.257
B_1_WD	0.884	−0.588	−1.680	0.029	1.816	3.299
Cracked samples						
CCRW	0.993	−0.151	0.121	0.000	0.245	0.060
B_1_B_2_CRW	0.747	−0.640	−2.353	0.034	3.444	11.860
B_2_CRWD	0.987	−0.569	0.015	−0.002	0.707	0.500
B_1_CRWD	0.846	1.979	3.935	1.666	4.189	17.545

Legend: chi-square statistic (*χ*^2^), root mean square error (RMSE), mean bias error (MBE), mean percentage error (MPE), sum of squared errors (SSE), average absolute relative deviation (AARD), along with additional statistical characteristics such as the coefficient of determination (*r*^2^), skewness (Skew), kurtosis (Kurt), mean, standard deviation (StDev), and variance (Var).

**Table 4:** Artificial neural network modeling.

Non-Cracked Samples						
Network Name	Training Performance	Test Performance	Validation Performance	Training Error	Test Error	Validation Error
MLP 5-3-1	0.937266	1.00000	1.00000	1.589114	1.705785	4.477483
	Training algorithm	Error function	Hidden activation		Output activation	
	BFGS 24	SOS	Exponential		Logistic	
ANN verification	*χ* ^2^	RMSE	MBE	MPE	SSE	AARD
	4.191	1.982	0.429	3.892	62.872	3.892
	*r* ^2^	Skew	Kurt	Mean	StDev	Var
	0.877	0.964	0.987	0.429	1.999	3.995
Cracked samples						
Network name	Training performance	Test performance	Validation performance	Training error	Test error	Validation error
MLP 5-5-1	0.915112	1.00000	1.00000	2.44003	1.107999	12.15342
	Training algorithm	Error function	Hidden activation		Output activation	
	BFGS 19	SOS	Tangent hyperbolic		Tangent hyperbolic	
ANN verification	*χ* ^2^	RMSE	MBE	MPE	SSE	AARD
	7.440	2.641	0.558	5.976	111.606	5.976
	*r* ^2^	Skew	Kurt	Mean	StDev	Var
	0.810	0.946	0.716	0.558	2.666	7.108

Legend: chi-square statistic (χ^2^), root mean square error (RMSE), mean bias error (MBE), mean percentage error (MPE), sum of squared errors (SSE), average absolute relative deviation (AARD), along with additional statistical characteristics such as the coefficient of determination (r^2^), skewness (Skew), kurtosis (Kurt), mean, standard deviation (StDev), and variance (Var).

## Data Availability

The original contributions presented in the study are included in the article, further inquiries can be directed to the corresponding authors.
